# Evaluating plasma adipokines and their cognate receptors as biomarkers for non-invasive diagnosis of endometrial cancer

**DOI:** 10.1042/BSR20253508

**Published:** 2025-09-22

**Authors:** Rebecca Karkia, Eshwa Zahra, Chaeyeoun Min, Kako Hirai, Evgeny Makarov, Emmanouil Karteris, Jayanta Chatterjee

**Affiliations:** 1 College of Health, Medicine and Life Sciences, Brunel University London, Uxbridge, UB8 3PH, U.K; 2 Academic Department of Gynaecological Oncology, Royal Surrey NHS Foundation Trust, Guildford, GU2 7XX, U.K

**Keywords:** adipokines, biomarkers, endometrial cancer, liquid biopsy

## Abstract

Endometrial cancer (EC) is the most common gynaecological malignancy in developed countries. Early detection remains challenging, with no established plasma-based biomarkers for clinical use. This study aimed to evaluate plasma adipokines and their receptor expression as diagnostic biomarkers for EC. Plasma levels of leptin, soluble leptin receptor, visfatin and asprosin were quantified in EC and control patients using ELISA. The free leptin index (FLI) was calculated as a ratio of leptin to soluble leptin receptor. Gene expression of corresponding receptors, including leptin receptor (*Ob-R*), insulin receptor (*INSR*), glucagon-like peptide-1 receptor [GLP-1 receptor (*GLP-1R*)], and asprosin-associated receptors, toll-like receptor 4 (*TLR4*), protein tyrosine phosphatase receptor type D (*PTPRD*), and olfactory receptor family 4 subfamily M member 1, was assessed by RT-qPCR from total blood. Plasma leptin levels were significantly elevated in EC patients, with the FLI over four times higher than controls (*P*=0.008). Soluble leptin receptor levels trended lower in EC, though non-significantly. Visfatin and asprosin plasma levels showed non-significant elevations. Gene expression analyses revealed significantly increased levels of *GLP-1R*, *TLR4* and *PTPRD* in EC patients, suggestive of a diagnostic potential. Notably, plasma biomarker levels were not independently correlated with body mass index (BMI). Elevated FLI and up-regulation of adipokine receptor expression highlight the potential of combining plasma-based and molecular biomarkers for EC diagnosis. However, the lack of independence from BMI and conflicting literature underscores the need for larger, standardised studies to validate these findings and determine clinical applicability.

## Introduction

In the UK, 9828 new cases of endometrial cancer (EC) were registered in 2017–2019 [1]. The incidence of endometrial hyperplasia is estimated to be at least three times higher than EC [2].

The most common presentation of endometrial hyperplasia and cancer is abnormal uterine bleeding. This includes heavy menstrual bleeding, intermenstrual bleeding, irregular bleeding, unscheduled bleeding on hormone replacement therapy (HRT) and post-menopausal bleeding (PMB). Whilst the most common decade of diagnosis of EC is in the seventh decade, there is an increasing trend towards diagnosis of younger pre-menopausal women [3].

Recent advances using high-throughput technologies for biomarker discovery based on genomic, transcriptomic, proteomic and metabolomic platforms have shown excellent potential for the non-invasive diagnosis of cancer in numerous fields [4–6]. However, at present, there is no routinely used biomarker for detection of cancer that has demonstrated similar performance to endometrial biopsy, the gold standard for EC detection. This limits the utilisation of EC screening techniques at the present time. Although promising results have emerged from studies investigating serum and plasma adipokines as diagnostic biomarkers, significant heterogeneity among these studies indicates that further evidence is required before they can be considered for clinical use [7–9].

Previous work from our group based on statistical meta-analysis of risk factors for EC has shown that women with body mass index (BMI) above 25 had a higher risk, with the risk increasing as BMI rises [10]. We have expanded on these findings, curating data from the UK Biobank in order to study in more detail the association between metabolic syndrome (MetS) and the risk of EC in pre- and post-menopausal women [11]. In this study that included 177,005 females, we have shown that certain components of MetS (including waist circumference, BMI, hypertension, hyperlipidaemia and diabetes) independently and in combination significantly increase the risk of EC. Interestingly, when pre- and post-menopausal subgroups were compared, diabetes and hypertriglyceridaemia were found to be the strongest predictors of EC in the pre-menopausal subgroup. We have proposed that a combination of diagnostic components such as MetS and obesity in the pre-menopausal population is potentially an important clinical differentiator for this cancer. Of note, other factors such as insulin resistance and hyperinsulinaemia have also been shown to be associated with increased risk for EC, independent of obesity [12]

Therefore, adiposity is arguably the most prominent hallmark of MetS, as it is associated with numerous metabolic abnormalities that are linked to the development of EC. Adipokines are peptides that are released from various types of adipose tissue and exert numerous effects in health and disease, as they regulate important physiological functions such as metabolism, inflammation and insulin resistance to name a few [13]. Their dysregulation contributes to disorders that are related to obesity, including cancer. For example, in hepatocellular carcinoma (HCC), circulating levels of adiponectin, leptin, visfatin and resistin were significantly higher in HCC patients when compared with the control group [14].

We have also performed a meta-analysis and systematic review on the diagnostic accuracy of liquid biopsies for EC and have shown that certain biomarkers, including visfatin and leptin, warrant further investigation [5]. We have decided to expand on these observations and include glucagon-like peptide-1 (GLP-1) given that GLP-1 receptor agonists can reduce cancer incidence, particularly in patients with type-2 diabetes. We have also included in this study measurements of the newly described adipokine, asprosin, given its role in various reproductive-related diseases including polycystic ovary syndrome (PCOS) and endometrial and ovarian cancer [15–17].

Therefore, the primary objective of this clinical study was to analyse the diagnostic performance of plasma adipokines such as leptin, visfatin and novel biomarkers asprosin and GLP-1. The idea was to identify a number of non-invasive candidate biomarkers that can be used to differentiate between EC, endometrial atypical hyperplasia (CAH) and benign endometrium in symptomatic women presenting with PMB. As a secondary objective, we have used the same liquid biopsies to measure expression of the cognate receptors of the above-mentioned cytokines in order to see whether they have a clinical utility as diagnostic biomarkers.

## Materials and methods

### Study setting and recruitment

This study was conducted at the Royal Surrey NHS Foundation Trust, a gynaecological oncology tertiary referral centre in Guildford, U.K, that performs approximately 200 EC surgeries annually. The centre serves a mixed patient population, with around 60% of EC cases referred by neighbouring cancer units. Approximately 500 patients present annually to the rapid access clinic for suspected EC based on PMB. Of these, 5–10% are diagnosed with EC, translating to around 25 referrals from the local unit per year for surgical management. The research has been carried out in accordance with the World Medical Association Declaration of Helsinki, and all subjects have provided informed consent.

### Inclusion and exclusion criteria

Participants were eligible for inclusion if they were:

- Female adults over 18 years old, capable of providing informed consent.
- Referred for investigation of PMB or peri-menopausal abnormal uterine bleeding.
- Considered suitable for routine clinical investigation by the principal investigator.

Participants were excluded if they had had:

- Previous hysterectomy or EC diagnosis.
- Concurrent non-EC or ongoing chemotherapy/radiotherapy.
- Declining endometrial biopsy during clinical assessment.
- Any condition compromising safety or data integrity.

### Ethical approval

Ethical approval for this study was granted on April 24, 2023, by the South West – Frenchay Research Ethics Committee (23/SW/0013). A material transfer agreement between the Royal Surrey NHS Foundation Trust and Brunel University was established.

### Recruitment

Recruitment of patients began in August 2023 and was completed in April 2024, with 103 venous blood samples collected. Recruitment targeted 50 EC patients and 50 controls without malignancy.

### Sample collection and handling

Venous blood (8 ml) was collected in EDTA or Streck tubes and refrigerated before same-day laboratory processing. Whole blood (0.5 ml) was stored in 1 ml RNAlater (Invitrogen) in labelled Eppendorf Tubes and frozen at −80°C. Plasma was separated by centrifugation at 2500×g for 10 minutes, aliquoted into 1 ml fractions, and stored at −80°C.

### RNA extraction

Total RNA was extracted from whole blood using the Maxwell® RSC simplyRNA Blood Kit (Promega). The procedure involved lysis, DNase treatment and automated purification using the Maxwell® RSC Instrument. Blood samples stabilised in RNAlater were thawed before RNA extraction. Extracted RNA was stored at −80°C until analysis.

### RNA quantification

RNA quality and concentration were assessed using the Quantifluor dsDNA Dye System and a Quantus Fluorometer (Promega). Samples with yields ranging from 1 to 50 ng/μl were deemed suitable for downstream analysis, with a median yield of 4 ng/μl.

### cDNA synthesis

Complementary DNA (cDNA) was synthesised using the High-Capacity cDNA Reverse Transcription Kit (ThermoFisher). A master mix containing random primers and reverse transcriptase was prepared, and 10 μl of undiluted RNA was used per 20 μl reaction. Thermal cycling conditions were 25°C for 10 minutes, 37°C for 120 minutes and 85°C for 5 minutes, followed by storage at −20°C.

### Primer design and validation

Primers were sourced from the Harvard Primer Bank and synthesised by Sigma Aldrich. The primer sequences were as specified in Table 1. Primer specificity was validated by performing conventional PCR on Ishikawa and RL95-2 EC cell lines, followed by agarose gel electrophoresis. Bands were analysed to confirm amplification of the expected product size before proceeding with RT-qPCR.

**Table 1:** Primer sequences

Primer	Forward primer	Reverse primer
YWHAZ	AGACGGAAGGTGCTGAGAAA	GAAGCATTGGGGATCAAGAA
B-Actin	CAAGATGAGATTGGATGGC	CACATTGTGAACTTTGGGG
TLR4	AGTTGATCTACCAAGCCTTGAGT	GCTGGTTGTCCCAAAATCACTTT
PTPRD	CAGGCGGAAGCGTTAATATCA	TTGGCATATCATCTTCAGGTGTC
OR4M1	TCTGTTAATGTCCTATGCCTTCC	AATGTGGGAATAGCAGGTGG
Leptin receptor	CATTTTATCCCCATTGAGAAGTA	CTGAAA ATTAAGTCCTTG TGCCCAG
GLP-1 receptor	TCGCTGTGAAAATGAGGAGG	TTGGCTGAGGTTAGAAGAGCC
Insulin receptor	AAAACGAGGCCCGAAGATTTC	GAGCCCATAGACCCGGAAG

### RT-qPCR

Gene expression analysis was performed on a QuantStudio 7 Flex Real-Time PCR System using SYBR™ Green PCR Master Mix. Each reaction was conducted in triplicate in a 96-well plate. Relative expression (RQ) values were calculated using the 2^(–ΔCt) method after normalization to the housekeeping gene β-actin. Ct values with intra-sample differences >1 were excluded from analysis. Differences in gene expression between EC cases and controls were graphically represented.

### Clinical study biomarker analysis: ELISA

The ELISA kits were purchased for the purposes of performing sandwich enzyme-linked immunosorbent assay on the study population to determine the difference in plasma adipokine levels between EC cases and controls. All kits purchased were pre-coated ELISA 96-well plates. These were: human leptin ELISA kit (Invitrogen, KAC2281), Quantikine Human Leptin R ELISA Kit (DOBR00), Human Asprosin ELISA kit (abx257694), Human Visfatin ELISA Kit (ab267658) and human GLP-1 ELISA (ab277395).

Asprosin ELISA was performed in accordance with manufacturer instructions. This ELISA is designed to detect 0.156–10 ng/ml asprosin with a sensitivity of <0.06 ng/ml. The first item to prepare is the standard solution. This was made with 1 ml of standard diluent buffer to make the 20 ng/ml standard solution. Starting with the standard solution at 20 ng/ml, the highest standard 10 ng/ml was made by further diluting by a factor of 2. Next, 0.5 ml of the highest standard solution was added to the 1st tube and mixed thoroughly. Next, 0.5 ml was transferred from the 1st to the 2nd tube and mixed thoroughly. The process was repeated until the final tube with a concentration of 0.16 ng/ml. The wash buffer was then reconstituted by diluting the concentrated wash buffer 30-fold with distilled water. The total volume of the working solution was calculated and then detection reagents A and B (as provided from the kit) were diluted with diluents A and B at a dilution of 1:100. Following this, 100 µl of diluted standard was aliquoted into the standard wells and, 100 µl of neat sample was then added in the remaining antibody-coated wells. The plate was then incubated for one hour at 37°C. Following incubation, 100 µl of detection reagent A was added to each well and incubated for a further one hour at 37°C. The plate was then washed three times with the wash buffer, with the contents discarded between washes. A total of 100 µl of detection reagent B was then added to each well and incubated at the same temperature for 30 minutes. A further five washes were performed with the wash buffer before adding 90 µl of the TMB substrate to each well. The plate was then incubated for 20 minutes at 37°C before adding 50 µl of the stop solution. The plate was then read on the microplate reader and the optical density (OD) measured spectrophotometrically at 450 nm. The OD readings for each reference standard and each sample were then calculated by subtracting the average control (zero) OD reading; i.e. [(relative OD) = (OD of each well) – (OD of zero well)]. The standard curve was then plotted as the relative OD of each reference standard solution (X), against the respective concentration of each standard solution (Y). Subsequently, the concentration of the samples was interpolated from the standard curve.

The GLP-1 ELISA was also conducted in accordance with the manufacturer’s instructions. The reagents, samples and standards were prepared as instructed. A total of 100 μL of standard or sample was added to each well. This was incubated for 2.5 hours at room temperature. The solution was then discarded, and the plate was washed four times with 300 μl 1X wash solution. A total of 100 μl of prepared biotin antibody was then added to each well and incubated for one hour at room temperature. A further four washes were conducted. A total of 100 μl prepared streptavidin solution was then added and left to incubate for 45 minutes at room temperature. After a repeat washing step, 100 μl TMB substrate reagent was added to each well and incubated for 30 minutes at room temperature. 50 μl stop solution was then added, and the plate was read at 450 nm immediately.

Similarly, the Leptin ELISA was conducted in accordance with the manufacturer’s instructions. For this study, a fifty-fold dilution of plasma was performed. The reagents, samples and standards were prepared as instructed. Absorbance of each well was read at 450 nm having blanked the plate reader against a chromogen blank composed of 100 µl each of stabilised chromogen and stop solution. Curve-fitting software was used to generate the standard curve. A four-parameter algorithm provided the best standard curve fit. Samples producing signals greater than that of the highest standard were excluded.

The soluble leptin receptor ELISA was conducted in accordance with the manufacturer’s instructions. The reagents, samples and standards were prepared as instructed. A five-fold dilution of the samples was performed. OD was plotted on the y-axis and plasma concentration on the x-axis. The line of best fit was found to be a four-parameter curve fit was found to be the best choice. After calculating the concentrations according to the non-linear equation, the resulting values were multiplied by the sample dilution factor. Samples producing signals greater than that of the highest standard were excluded.

Finally, the Visfatin ELISA was also conducted in accordance with the manufacturer’s instructions. The reagents, samples and standards were prepared as instructed. No dilution of the samples was performed. Absorbance of each well was read at 450 nm. The standard curve was plotted on log-log, with standard concentration on the x-axis and absorbance on the y-axis.

### Statistical analysis

All statistical analyses were performed using GraphPad Prism version 9. Continuous variables were expressed as mean ± standard deviation (SD) or median (interquartile range, IQR), as appropriate. Categorical variables were presented as frequencies and percentages.

Comparisons between groups were made using the Student’s t-test or Mann–Whitney U test for continuous variables and the chi-square test or Fisher’s exact test for categorical variables. The diagnostic performance of biomarkers was assessed using receiver operating characteristic (ROC) curve analysis, and the area under the curve (AUC) was calculated to evaluate the accuracy of each biomarker. Scatterplots were generated to visualise the relationship between plasma adipokine levels and potentially important co-variates such as age and BMI. Spearman’s R correlation test was used to assess the association between these variables.

## Results

### Description of baseline characteristics

A total of 103 patients were recruited to the study, completing the target accrual (Table 2). The baseline characteristics of the study population, including the control group (*N*=53), EC group (*n*=47) and AH group (*n*=3), reveal notable differences across demographic, clinical and histological variables. The median age of participants was significantly higher in the EC group (69.0 years; IQR: 59.0–75.0) compared with the control group (57.0 years; IQR: 53.0–62.0) (*P*=0.2). The AH group had a median age of 67.0 years (IQR: 63.0–70.5). Ethnically, the majority of participants in both the control and EC groups were Caucasian (94.3% and 100.0%, respectively), with no significant difference between groups (*P*=0.29). A small proportion of Asian individuals were present in both groups, and only one participant in the control group identified as Black/Afro-Caribbean. The EC group demonstrated a significantly higher median BMI (33.3; IQR: 26.6–38.2) compared with the control group (26.3; IQR: 22.8–32.4) (*P*=0.2). Participants in the AH group exhibited the highest median BMI (43.0; IQR: 38.0–46.7). Regarding smoking status, most participants in both the control (66.7%) and EC (55.3%) groups were never-smokers, with no significant difference between groups (*P*>0.05). Nulliparity was similar between the control (20.4%) and EC groups (17.7%) (*P*>0.05). Prior use of combined oral contraceptives was less frequent in the EC group (61.7%) compared with the control group (74.1%), though this difference was not statistically significant (*P*=0.15). HRT use was significantly less common in the EC group (23.4%) compared with the control group (66.0%) (*P*<0.001). Hypertension was significantly more prevalent in the EC group (48.9%) compared with the control group (20.4%) (*P*=0.002). Other medical conditions, such as diabetes, hypercholesterolaemia, asthma and hypothyroidism did not differ significantly between groups (*P*>0.05). Notably, breast cancer and tamoxifen use were uncommon but were observed with similar frequencies between groups. Among EC cases, the most common histological subtype was endometrioid adenocarcinoma (74.5%), followed by serous carcinoma and malignant mixed Müllerian tumours (10.6% each). Clear cell carcinoma and serous endometrial intraepithelial carcinoma each accounted for 2.1% of cases. The majority of EC cases were diagnosed at stage I (72.3%), with fewer cases presenting at stage II (10.6%) or stage III (17.0%). No cases were identified at stage IV.

**Table 2:** Baseline characteristics table of patients recruited to clinical study

Characteristics	Control group *N*=53	EC group *N*=47	Atypical hyperplasia *N*=3	*P*-value (control V EC)
Age; median (IQR)	57.0 (53.0–62.0)	69.0 (59.0–75.0)	67.0 (63.0–70.5)	<0.001
Ethnicity, N (%)				
Caucasian	50 (94)	47 (100)	3 (100)	0.25
Asian	2 (4)	0		0.65
Black/Afro-Caribbean, n (%)	1 (2)	0		0.33
BMI (Kg/m^2^); Median (IQR)	26.3 (22.8–32.4)	33.3 (26.6–38.2)	43.0 (38.0–46.7)	<0.001
Smoking, N (%)				
Never	36 (66.7)	26 (55.3)	2 (66.7)	0.24
Ex	17 (31.5)	16 (34.0)	1 (33.3)	0.8
Current	1 (1.9)	4 (8.5)	0 (0.0)	0.18
Nulliparous, N (%)	11 (20.4)	8 (17.7)	0 (0.0)	0.73
Menarche; median (IQR)				
Pre-menopausal; N (%)				
Previous COCP use; N (%)	40 (74.1)	29 (61.7)	3 (100.0)	0.15
HRT use (ever); N (%)	35 (66.0)	11 (23.4)	0	<0.001
Medical conditions; N (%)				
Diabetes	4 (7.5)	7 (14.9)	1 (33.3)	0.21
PCOS	3 (5.7)	1 (2.1)	0	0.41
HTN	11 (20.4)	23 (48.9)	1 (33.3)	0.002
Hypercholesterolaemia	4 (7.5)	3 (6.4)	0 (0.0)	0.85
Asthma	4 (7.5)	3 (6.4)	1 (33.3)	0.85
Hypothyroidism	2 (3.7)	4 (8.5)	0 (0.0)	0.4
Breast cancer	3 (5.6)	2 (4.3)	2 (66.7)	0.75
Tamoxifen use	2 (3.8)	2 (4.3)	2 (66.7)	0.89
Histological subtype				
Endometroid	-	35 (74.5)	-	
Serous	-	5 (10.6)	-	
MMMT	-	5 (10.6)	-	
Clear cell	-	1 (2.1)	-	
SEIC	-	1 (2.1)	-	
Stage				
1	-	34 (72.3)	-	
2	-	5 (10.6)	-	
3	-	8 (17.0)	-	
4	-	0 (0.0)	-	
Grade				
1	-	23 (48.9)	-	
2	-	8 (17.0)	-	
3	-	16 (34.0)	-	

BMI, body mass index. COCP, combined oral contraceptive pill. HRT, hormone replacement therapy. HTN, hypertension. PCOS, polycystic ovary syndrome. MMMT, malignant mixed Mullerian tumour. SEIC, serous endometrial intraepithelial carcinoma.

### Circulating leptin levels and free leptin index (FLI)

**Figure 1:**
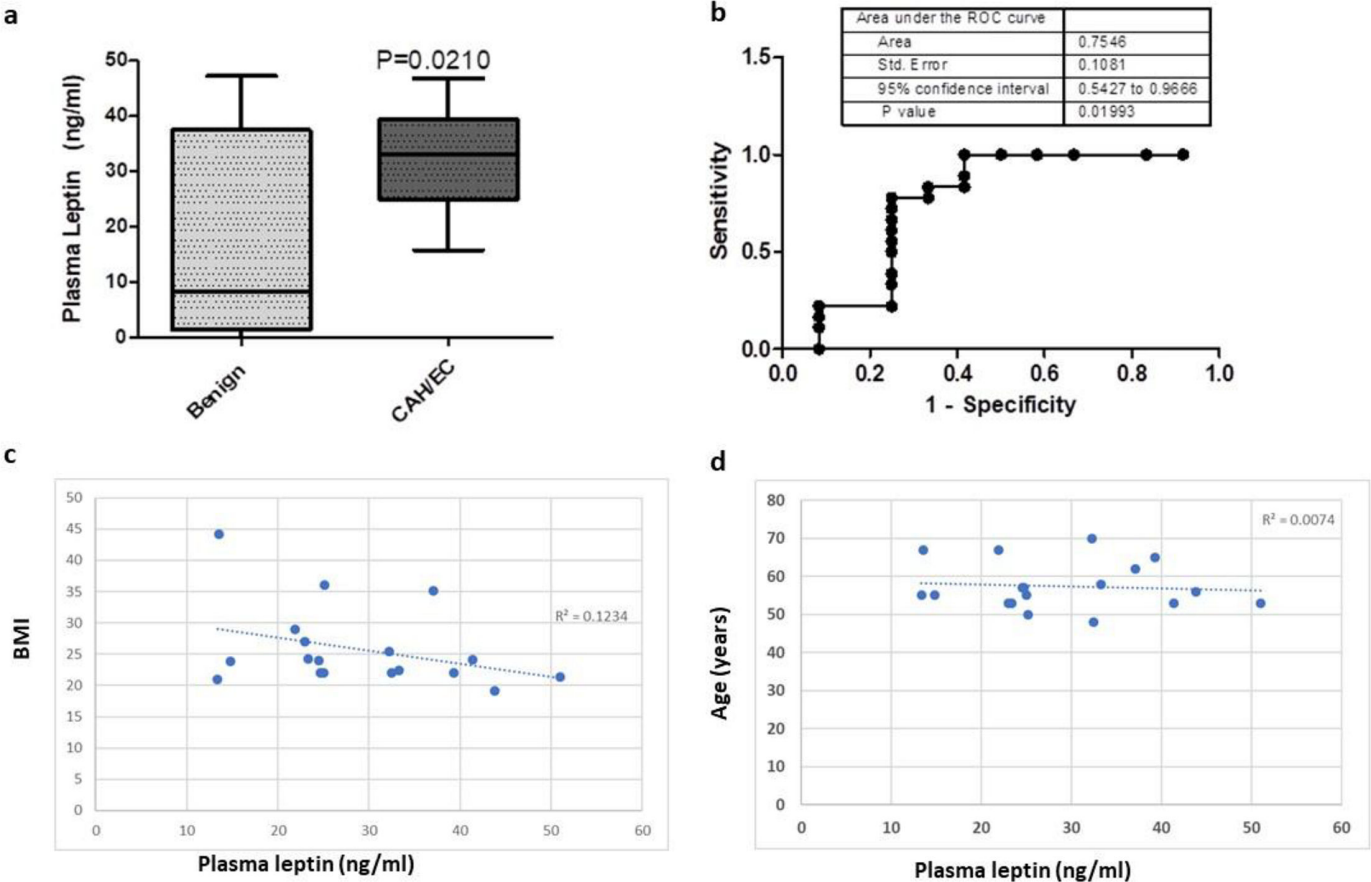
Leptin levels in EC patients. (**a**) Box‐and‐whisker plots of plasma leptin levels (ng/ml) in benign controls (*n*=12) versus CAH/EC patients (*n*=18). Boxes extend from the 25th to the 75th percentile, with the horizontal line indicating the median; whiskers denote the minimum and maximum values. Median plasma leptin concentrations were 8.23 ng/ml in the benign group and 33.06 ng/ml in the CAH/EC group, reflecting a statistically significant difference (*P*=0.021). (**b**) ROC curve for plasma leptin levels distinguishing benign from CAH/EC patients. The area under the curve (AUC) is 0.7546 (SE=0.1081; 95% CI: 0.5427–0.9668; *P*=0.019), demonstrating moderate diagnostic accuracy. (**c**) Scatter plot showing the relationship between BMI and plasma leptin concentrations in individual patients. Each point represents a single benign participant’s measurements. The R^2^ correlation coefficient indicated no strong association between BMI and leptin levels in this cohort. (**d**) Similarly, a scatter plot illustrating the relationship between age and plasma leptin concentrations did not produce any strong association between these variables.

A 96-well ELISA was conducted assessing plasma levels of leptin in 40 patients. After standard curve corrections, 10 patients were excluded from analysis as results were off-curve low or undetectable. In total, 12 patients with benign endometrial changes had a median plasma level of 8.2 ng/ml (IQR: 1.5–37.5) and 18 patients with AH/EC had a median plasma level of 33.1 ng/ml (25.0–39.5) (Figure 1a). The difference between the two groups was statistically significant (*P*=0.021). The AUC of leptin as a diagnostic marker was 0.754 (95% CI: 0.543–0.967) (Figure 1b). To assess whether significance was due to confounding factors such as age or BMI, linear regression was used to plot age or BMI against the leptin concentration in the control cohort. Neither age nor BMI was significantly associated with leptin concentration in benign patients as determined by Spearman’s R correlation coefficient (Figure 1c–d).

**Figure 2:**
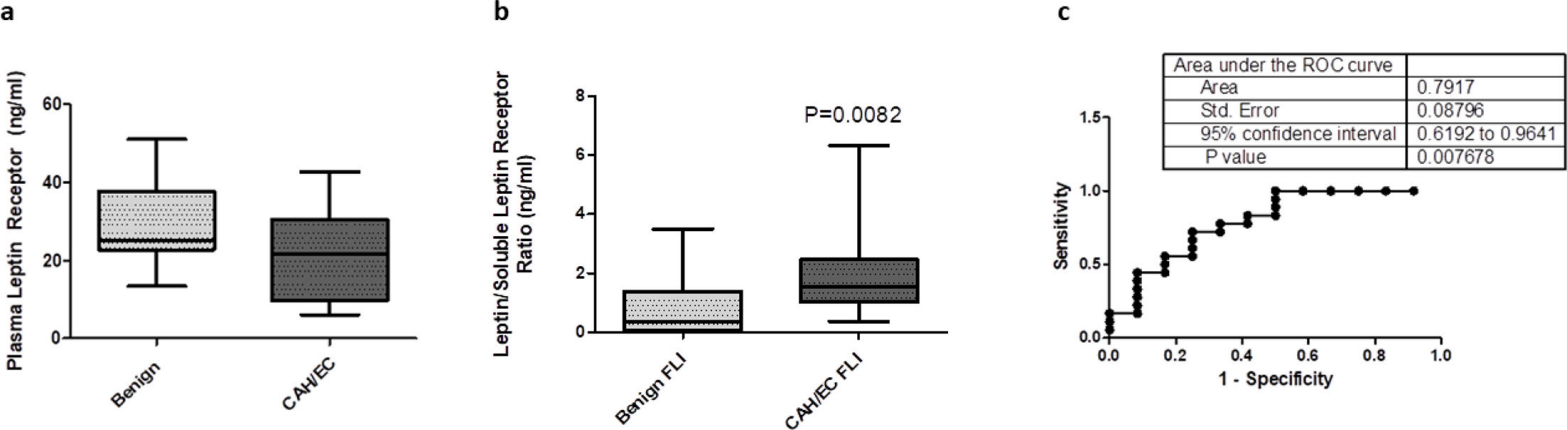
Soluble leptin receptor levels and free leptin index (FLI). (**a**) Box‐and‐whisker plots of plasma leptin receptor levels (mg/ml) in benign controls (*n*=18) versus CAH/EC patients (*n*=22). Boxes span the 25th to 75th percentile, with the horizontal line indicating the median and whiskers extending to the minimum and maximum observed values. The difference between groups did not reach statistical significance (*P*=0.071). (**b**) Box‐and‐whisker plots of the leptin/soluble leptin receptor ratio (ng/ml; FLI) in benign controls (*n*=12) versus CAH/EC patients (*n*=18). The CAH/EC group shows a significantly higher ratio (*P*=0.0082). (**c**) Receiver operating characteristic (ROC) curve for the leptin/soluble leptin receptor ratio in differentiating benign controls from CAH/EC patients. The area under the curve (AUC) is 0.7917 (SE=0.08796; 95% CI: 0.6192–0.9641; *P*=0.0076), indicating a statistically significant diagnostic performance for this ratio

Plasma levels of soluble leptin receptor were also assessed in 18 patients with benign disease and 22 with EC. The median plasma soluble leptin receptor level was higher at 25.1 ng/ml as compared with 21.6 ng/ml in CAH and EC patients; however, this was not statistically significant (*P*=0.071, Figure 2a). Subsequently, the free leptin index (FLI) was measured by dividing whole plasma leptin levels by the soluble plasma leptin receptor levels in each patient and then comparing EC cases and controls. The FLI was found to be over four times higher in EC patients as compared with control. The median FLI in cases was 0.36 ng/ml (95% CI: 0.15–1.47) versus 1.5 ng/ml (95% CI: 1.26–2.94), *P*=0.008 (Figure 2b). The AUC of the FLI as a diagnostic marker for EC was 0.792 (95% CI: 0.619–0.964) (Figure 2c).

### Circulating visfatin, GLP-1 and asprosin levels in EC patients

**Figure 3:**
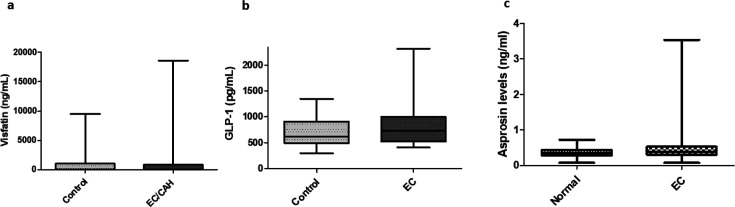
Box‐and‐whisker plots of plasma visfatin levels (ng/ml). (**a**) GLP-1 (pg/ml) (**b**), and asprosin (ng/ml) (**c**) in benign controls versus EC/CAH patients. There was no statistically significant difference in the circulating levels amongst these groups.

Similarly, plasma visfatin levels were assessed in 18 control patients and 22 EC patients. The median visfatin level in benign patients was 11.0 ng/ml (IQR: 2.3–1020.0) versus 14.5 ng/ml in controls (IQR: 2.6–829.3), *P*=0.817 (Figure 3a). The median GLP-1 level in benign patients was 613.6 pg/ml (IQR: 493.4–907.7 pg/ml) versus 729.0 pg/ml (IQR: 524.5–995.9 pg/ml), *P*=0.334 (Figure 3b). Finally, the median asprosin level in benign patients was 0.35 ng/ml (IQR:0.28–0.44) versus 0.38 ng/ml in EC patients (IQR:0.31–0.52), *P*=0.4010 (Figure 3c).

### Expression of leptin, insulin, GLP-1 and putative asprosin receptors in whole blood

**Figure 4:**
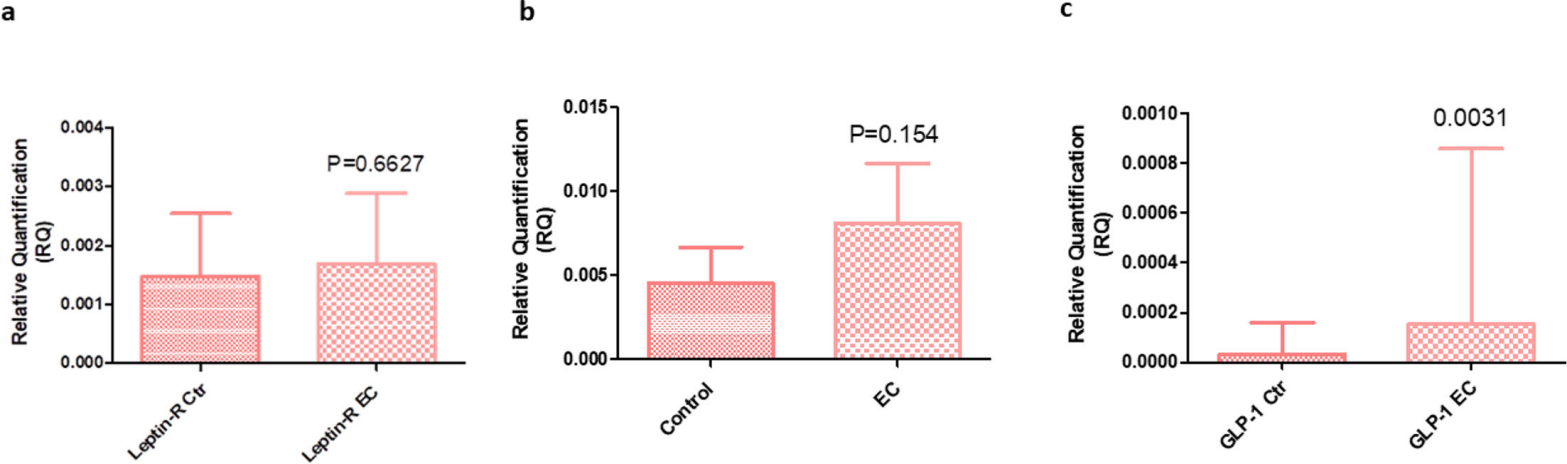
Expression of *LEPR*, *INSR* and *GLP-1R*. (**a**) Relative quantification (RQ) of leptin receptor expression (*LEPR*) in total blood from EC patients (*n*=36) compared with controls (*n*=32), *P*=0.6627. (**b**) RQ of insulin receptor expression (*INSR*) in total blood from EC patients (*n*=30) compared with controls (*n*=27), *P*=0.154. (**c**) RQ of *FLP-1R* expression in total blood from EC patients (*n*=26) compared with controls (*n*=25), *P*=0.0031.

Following measurement of circulating adipokine levels, we assessed the gene expression of their putative receptors in whole blood in an attempt to identify if any of them could be a biomarker of potential clinical utility. There were no major changes in the expression of leptin receptor (*Ob-R*) compared in patients with AEH/EC versus control patients, as assessed by RT-qPCR (*P*=0.663, Figure 4a). Similarly, when insulin receptor (*INSR*) expression, the known receptor of visfatin, was compared in patients with EC versus control patients with no malignancy, there was a trend towards increased insulin receptor expression, but no significant difference was found (*P*=0.154, Figure 4b). Interestingly, there was a significant increase in GLP-1 receptor (*GLP-1R*) expression in the cancer cohort (*P*=0.0031; Figure 4c).

**Figure 5:**
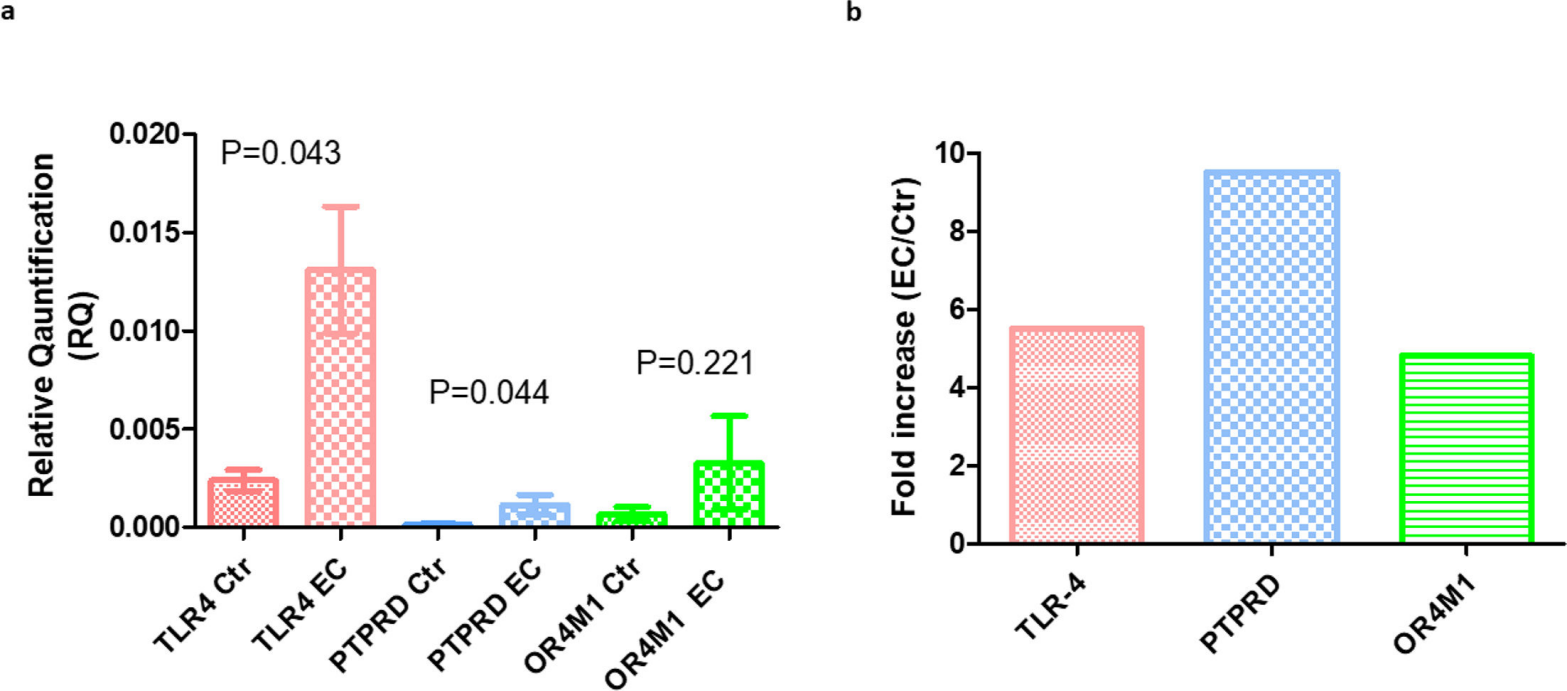
RQ of *TLR4*, *PTPRD* and *OR4M1* in EC patients versus controls, quantified by RT‐qPCR. (**a**). cDNA was synthesised from extracted RNA, and each patient’s sample was run in triplicate. Bars represent mean ± SEM, and the Mann–Whitney U test was used to assess significance. TLR4 (*P*=0.043) and PTPRD (*P*=0.044) were significantly elevated in EC compared with controls, whereas OR4M1 levels did not differ between groups (*P*=0.221). The total number of samples analysed was *n*=57 for *TLR4*, *n*=50 for *PTPRD* and *n*=36 for *OR4M1*. The fold increase in the expression (EC/Ctr) for TLR-4 was 5.5, for PTPRD 9.5 and for OR4M1 was 4.8 (**b**).

Gene expression of the three proposed receptors for asprosin was also assessed in patients with EC versus control patients with no malignancy (Figure 5a–b). Relative toll-like receptor 4 (*TLR4)* gene expression was evaluated in 23 control patients and 32 EC patients. There was a significant difference in the relative gene expression between the groups, with EC patients tending towards higher expression of *TLR4* (*P*=0.043). No significant changes in the expression of protein tyrosine phosphatase receptor type D (*PTPRD)* and olfactory receptor family 4 subfamily M member 1 (*OR4M1)* were observed amongst those with EC versus non-cancer controls (*P*=0.044 and *P*=0.221, respectively).

## Discussion

In this study, we explored whether plasma-based adipokines and their receptors have a clinical utility as diagnostic biomarkers for EC. Results from this study show that plasma leptin levels were significantly higher in patients with AH and EC than in benign control patients. When the FLI was calculated by dividing total leptin from the soluble leptin receptor levels, the FLI was over four times higher in EC patients as compared with controls. Our findings of increased plasma leptin levels in EC patients are consistent with the literature, where overall trends towards higher leptin levels were seen in EC patients when compared with benign controls. In a meta-analysis by Wang et al., six included studies showed that raised leptin was associated with a significantly increased relative risk of EC development [18].

However, there are a number of discrepancies in the reported literature both in terms of the reported serum/plasma ranges and also when controlling for confounding factors. For example, in a study by Petridou et al., the serum leptin levels were determined in fasting morning blood samples. The mean serum leptin levels were 36.7 +/- (SD) 25.7 ng/ml among cases and 26.9 ± 19.8 ng/ml in controls. Whilst the odds ratio for an increment of 1 SD of blood leptin was 1.52 before adjustment for BMI, after adjustment there was no significant difference with an odds ratio (OR) of 1.13 [19]. Similar findings were reported in a study of 146 post-menopausal females with EC and 150 control subjects. Authors found the mean levels of leptin in EC cases were double that of controls (8.2 versus 4.5 ng/ml *P*<0.0001 respectively). They too found a significant correlation with BMI, concluding therefore that leptin is not independently correlated with EC risk [20]. In contrast, the study by Ma et al. which examined serum levels in 516 cases and controls reported higher serum leptin concentrations than controls (28.8 ± 2.2 ng/ml versus 19.8 ± 1.4 ng/ml). Stratification by menopausal status, parity, HRT use or diabetes status was also performed, and leptin levels remained a significant predictor of EC risk after control for these potential confounding factors [21].

In our study, although the number of patients was small, there was no significant correlation between the leptin concentrations and BMI. It is unclear, given limited data and conflicting information from published studies, whether such a correlation truly exists. In a more recent meta-analysis analysing the effect of weight loss on leptin levels, 20 eligible studies were assessed and reported an overall average of 40% reduction in leptin levels associated with weight loss [22]. Whilst the literature may be consistent in reporting that leptin is found in higher concentrations in those patients with EC versus non-cancer controls, this does not appear to be independent of BMI after all, suggesting that leptin may not be a useful standalone diagnostic biomarker.

In serum, soluble ObRe modulates the leptin bioavailability and has been shown to be decreased in obese humans [23]. In breast cancer patients, soluble ObRe is overexpressed independently of the oestrogen receptor status [24]. Furthermore, the ratio between leptin/ObR serum levels and the FLI is considered a useful predictor of leptin activity and has been shown to be increased in cases of breast cancer development [25]. In this matched pairs case-control study, Rodrigo et al. recruited 80 patients with sporadic breast cancer and matched them with 80 healthy controls (matched for age, BMI and menopausal status). The results showed that leptin, leptin/BMI ratio, FLI and visfatin were significantly higher and soluble leptin receptor ObRe was significantly lower in patients with breast cancer [25]. In the study by Mohammadzadeh et al [26], median serum levels of sOB-R in controls were significantly higher than those in breast cancer cases versus controls. Conversely, the median serum level of leptin in breast cancer cases was significantly higher than that in controls. Moreover, an increased FLI was significantly associated with breast cancer [26]. Similar trends have been demonstrated between soluble leptin receptor and risk of colorectal cancer [27]. There have been few studies of soluble leptin and the FLI in EC, with one Mendelian randomisation study failing to find any causal association of soluble leptin and EC [28].

No major differences were evident in the circulating visfatin levels or its receptor expression in EC samples compared with healthy controls. A previous small study looking at 42 EC cases and control patients found the average concentration of serum visfatin to be higher in EC patients [29]. Of note, this trend and the absolute levels were similar to those reported in our study. Authors also correlated visfatin concentration with degree of myometrial invasion, suggesting a cut-off of 26.8 ng/ml for optimal prediction. As mentioned before, Rodrigo et al. presented data with visfatin being significantly higher in patients affected with breast cancer when compared with controls matched for age, BMI and menopausal status [25]. It was also suggested that visfatin is involved in malignant progression mainly via INSR and PI3K/AKT and MAPK/ERK signalling pathways [29,30].

Similarly, plasma GLP-1 levels were marginally raised in the EC cohort as compared with the benign cohort, but the difference was not significant overall. There was, however, a significant increase in the expression of GLP-1R in EC patients compared with controls in total blood. GLP-1 is not an adipocytokine but an incretin hormone which has a number of synergistic and counterregulatory effects to the adipocytokines. For example, whereas GLP-1 stimulates pancreatic β-cells to secrete insulin, leptin has the counter effect, inhibiting insulin secretion [31]. Leptin tends towards pro-inflammatory effects, activating immune cells and contributing to inflammation, especially in adipose tissue. The contrary is thought to be true of GLP-1, as it has anti-inflammatory actions on peripheral tissues. Both GLP-1 and leptin synergistically enhance each other’s effects on reducing appetite and improving insulin sensitivity. In some studies, GLP-1R agonists can enhance the effectiveness of leptin, thereby improving its ability to suppress appetite [32]. *In silico* analysis of *GLP-1R* has shown that it is significantly increased in EC versus control patients in an analysis of TGCA data from GTEx (data not shown).

To date, there have been no published clinical studies examining GLP-1R’s potential role as a diagnostic marker. In a study investigating the effects of the GLP-1R agonist liraglutide in EC, liraglutide was found to inhibit Ishikawa cell growth in a dose-dependent manner [33]. Immunohistochemical analysis suggested GLP-1R expression was also associated with positive oestrogen receptor and progesterone receptor status, and higher GLP-1R expression was significantly correlated with better progression-free survival [33]. Overall, the receptor expression of GLP1 and its role in EC diagnostics appears to be promising, but at the present time, there is very little published data with the majority of studies being too small to draw firm conclusions.

Findings from this clinical study indicated that plasma asprosin levels were marginally higher amongst the EC cohort, but this was not statistically or clinically significant. Whilst there has been a significant variance in the reported levels of serum and plasma asprosin quoted in the literature, the values reported in this study were in the lower range. The reason for this is unclear, as the study group on the whole trended towards higher BMIs, a feature that is associated with higher asprosin levels. As with the other adipocytokines, it is likely that asprosin secretion is affected by feeding/fasting status and circadian rhythms, and thus study of receptor expression may be more informative and clinically useful in the study and search of a biomarker. The cognate receptor for asprosin is as yet unproven due to the relative paucity of research on asprosin. However, gene expression of the candidate receptors *TLR4*, *PTPRD* and *OR4MI* indicated that both *TLR4* and *PTPRD* were significantly up-regulated in the EC cohort as compared with controls. At the present time, no other group has examined the role of asprosin in EC, and thus there is very little available literature. However, as previously mentioned, asprosin has been studied in depth in relation to diabetes mellitus, PCOS and menopausal status. For this reason, further study into the role of asprosin in EC at the transcriptomic/metabolomic level using *in vitro* models will be of value. We also acknowledge further limitations of this study; e.g. addition of more controls could have better aligned the median age with that of the EC group. This is a significant limitation, and future work should include a larger control and patient cohort and further interrogation of liquid biopsies using a wider repertoire of techniques, including mass spectrometry, that may also reveal differentially expressed secretory proteins between control and EC groups that could have potential diagnostic value. Moreover, we plan to assess the expression of asprosin’s putative receptors using spatial transcriptomics using tumour microarrays (TMA)s. In terms of *in vitro* work, we will use siRNA or CRISPR-Cas9 to knock out PTPRD, TLR4 and OR4M1 in the future in order to define which is the main receptor that mediates these effects in EC.

## Conclusion

This study has explored the potential of plasma-based biomarkers and receptor gene expression analyses as diagnostic tools for EC. Elevated plasma leptin levels and a significantly higher FLI in EC patients highlight the relevance of adipokines in EC diagnostics. However, inconsistencies in the literature regarding leptin’s independence from BMI suggest it may not serve as a standalone biomarker. The focus on receptor expression, particularly soluble leptin receptor and transmembrane leptin receptor (Ob-R), provides a more stable alternative, though findings require further validation. Visfatin showed trends towards elevated levels in EC patients, and its association with cancer-related signalling pathways like PI3K/AKT and MAPK/ERK underscores its potential role in EC, though statistical significance was not achieved. GLP-1 receptor expression was significantly elevated in EC cases, suggesting its dual role as a diagnostic marker and therapeutic target. Additionally, asprosin’s receptor gene expression revealed significant up-regulation of *TLR4* and *PTPRD* in EC patients, highlighting multiple receptor-mediated mechanisms in this malignancy. These findings collectively emphasise the promise of adipokines and their receptors in advancing non-invasive diagnostic strategies for EC. However, larger, standardised studies are necessary to validate these results and establish their clinical utility in liquid biopsies. This work provides a foundation for integrating molecular and plasma-based approaches into EC diagnostics, addressing a critical gap in early detection and risk.

## Data Availability

Datasets are available on request. The raw data supporting the conclusions of this article will be made available by the authors, without undue reservation.

## Abbreviations

AH: atypical hyperplasia
AUC: area under the curve
BMI: body mass index
EC: endometrial cancer
FLI: free leptin index
GLP-1: glucagon-like peptide-1
GLP-1 receptor: glucagon-like peptide-1 receptor
HCC: hepatocellular carcinoma
HRT: hormone replacement therapy
IQR: interquartile range
MetS: metabolic syndrome
OD: optical density
OR4M1: olfactory receptor family 4 subfamily M member 1
PCOS: polycystic ovary syndrome
PMB: post-menopausal bleeding
PTPRD: protein tyrosine phosphatase receptor type D
ROC: receiver operating characteristic
RQ: relative quantification
SD: standard deviation
TLR4: toll-like receptor 4
cDNA: complementary DNA
